# c-Fos is a mechanosensor that regulates inflammatory responses and lung barrier dysfunction during ventilator-induced acute lung injury

**DOI:** 10.1186/s12890-021-01801-2

**Published:** 2022-01-06

**Authors:** Leilei Zhou, Chunju Xue, Zongyu Chen, Wenqing Jiang, Shuang He, Xianming Zhang

**Affiliations:** 1 School of Clinical Medicine, Guizhou Medical University, 550004 Guiyang, China; 2 Department of Respiratory and Critical Care Medicine, The Affiliated Hospital of Guizhou Medical University, 550004 Guiyang, China

**Keywords:** Acute respiratory distress syndrome, Ventilator-induced acute lung injury, c-Fos, T-5224, Fas/Fasl

## Abstract

**Background:**

As one of the basic treatments performed in the intensive care unit, mechanical ventilation can cause ventilator-induced acute lung injury (VILI). The typical features of VILI are an uncontrolled inflammatory response and impaired lung barrier function; however, its pathogenesis is not fully understood, and c-Fos protein is activated under mechanical stress. c-Fos/activating protein-1 (AP-1) plays a role by binding to AP-1 within the promoter region, which promotes inflammation and apoptosis. T-5224 is a specific inhibitor of c-Fos/AP-1, that controls the gene expression of many proinflammatory cytokines. This study investigated whether T-5224 attenuates VILI in rats by inhibiting inflammation and apoptosis.

**Methods:**

The SD rats were divided into six groups: a control group, low tidal volume group, high tidal volume group, DMSO group, T-5224 group (low concentration), and T-5224 group (high concentration). After 3 h, the pathological damage, c-Fos protein expression, inflammatory reaction and apoptosis degree of lung tissue in each group were detected.

**Results:**

c-Fos protein expression was increased within the lung tissue of VILI rats, and the pathological damage degree, inflammatory reaction and apoptosis in the lung tissue of VILI rats were significantly increased; T-5224 inhibited c-Fos protein expression in lung tissues, and T-5224 inhibit the inflammatory reaction and apoptosis of lung tissue by regulating the Fas/Fasl pathway.

**Conclusions:**

c-Fos is a regulatory factor during ventilator-induced acute lung injury, and the inhibition of its expression has a protective effect. Which is associated with the antiinflammatory and antiapoptotic effects of T-5224.

## Introduction

Mechanical ventilation (MV) is an important supportive treatment for severe diseases, such as acute respiratory distress syndrome. Although MV provides effective respiratory support treatment, improper use of mechanical ventilators can also induce lung injury or aggravate the original lung injury, resulting in ventilator-induced acute lung injury (VILI), which may cause an increase in mortality [1–4]. VILI includes volutrauma, barotrauma, and atelectotrauma; however, these conditions are all biological injuries in nature and are primarily characterized by pulmonary inflammation and impaired barrier function. Previous studies have confirmed that the possible targets of VILI are neutrophils, alveolar macrophages, alveolar epithelial cells, and reactive oxygen species; Neutrophils are the most important inflammatory cells in acute inflammation, and neutrophils are excessively activated and infiltrate the alveoli, secreting a large amount of cytotoxic substances and proinflammatory mediators, aggravating inflammation, damaging the alveoli and inhibiting neutrophils, which has been proven to reduce VILI in rabbits [5]. Alveolar macrophages are the main source of cytokines in the lungs. MV can quickly activate M1 macrophages. M1 macrophages release tumor necrosis factor-α (TNF-α) and interleukin-6 (IL-6), aggravating the inflammatory response. Macrophage polarization will also recruit a large number of peripheral neutrophils to the lungs, aggravating the inflammatory lesions of the lungs. In addition, the increase macrophages is related to pulmonary fibrosis and the impairment of pulmonary barrier function. Yin confirmed that interfering with M1 polarization of alveolar macrophages can significantly improve VILI [6, 7]. MV can directly stimulate the production of Reactive oxygen species (ROS) in vivo and in vitro and activate inflammatory cells to produce ROS, resulting in oxidative stress. Oxidative stress can directly damage deoxyribonucleic acid and protein through lipid peroxidation, and aggravate lung injuries by activating NF-κB to promote the release of cytokines. Some studies have confirmed that treatment with the antioxidant N-acetylcysteine can significantly reduce VILI [8, 9]. In addition, MV can cause calcium imbalance in alveolar epithelial cells, activate IL-8, TGF-β, etc., cause VILI. In addition, excessive apoptosis of alveolar epithelial cells during mechanical ventilation can cause damage to the lung barrier, aggravate lung injury, inhibit apoptosis, and reduce lung injury [10]. However, provision of interventions alone on these targets has little therapeutic effect [4, 11–13]. At present, no pharmacological method is available to alleviate VILI, and the most critical factors and signaling pathways that cause VILI have not been identified.

In recent years, with the in-depth study of c-Fos protein, researchers have found that c-Fos protein affects the occurrence and development of many diseases by controlling inflammation and apoptosis. c-Fos is an immediate early-response gene, that contains a stretch-sensitive promoter sequence that is expressed at low levels in the physiological state [14]. The c-Fos gene is highly conserved and can be rapidly and highly expressed under mechanical stress and other stimuli, which is related to the process of gene transcription, apoptosis, or proliferation. The function of the c-Fos gene is recognized by its encoded c-Fos protein. c-Fos protein and c-Jun proteins can specifically bind through the leucine zipper pathway to form transcription factor active protein1 (AP-1) with a heterodimer structure. AP-1 is associated with the occurrence and development of a variety of diseases through its promotion of inflammation and apoptosis, c-Fos/AP-1 directly controls the expression of inflammatory cytokines by binding to AP-1 motifs in the promoters of these genes; Lauricella shows that c-Fos could also directly band to the FasL promoter through a single AP-1 binding site in transient transfection experiments [15–19]. At present, c-Fos protein has made important progress in cell life activities, especially the correlation between the expression of c-Fos protein stimulated by various stress responses and some diseases [20–24].

The role and mechanism of c-Fos protein activation in VILI is well known. Tremblay et al. [25] observed that c-Fos RNA expression in isolated rat lung tissue increased under traumatic mechanical ventilation. Ying et al. [26] confirmed in vitro that c-Fos expression in alveolar epithelial cells increased under stress. Thus, c-Fos/AP-1 may play a vital role in the occurrence of VILI. Based on the crystal domain of the AP-1-DNA complex, Tsuhida et al. [27] synthesized the c-Fos/AP-1 specific inhibitor T-5224. T-5224 can regulate the gene expression of a variety of inflammatory cytokines and apoptotic proteins. In light of this research, we investigated whether c-Fos activation promote the progression of lung injury following injurious mechanical ventilation and whether inhibition of c-Fos protein may be a novel therapeutic strategy for VILI.

## Method

### Animal experiment

Thirty-six specific-pathogen-free (SPF) male Sprague–Dawley (SD) rats, aged 6–8 weeks and weighing 200–250 g, were purchased from Liaoning Changsheng Biotechnology Co. Ltd (Liaoning, China). (laboratory animal certificate number: SCXK (Liao) 2020-0001). The rats were housed in an SPF Animal Experimental Center, under 12-h light/dark cycle, and provided with food and drinking water ad libitum; however, they were fasted but provided with free drinking water 12 h prior to the experiment. The rats were intraperitoneally injected with 10% chloral hydrate (0. 35 ml/100 g) in the supine position and fixed on an adjustable thermal insulation pad. The neck skin was prepared. A 1 cm midline small incision was made, the fascia layers were separated, the thyroid gland was dissociated, blunt separation of cervical anterior muscle was performed using hemostatic forceps, the white trachea with circular cartilage was exposed, a 1-mm orifice was created on the neck that allows direct visualization of the trachea, a 0. 5–1 cm long polyethylene hose was inserted in the direction of the trachea, the hose was fixed in place, and breathing was kept unobstructed. The rats were then connected to a small animal ventilator (Chengdu Taimeng Software Co. Ltd, HX-100E, China). The animals were divided into six groups: a control group (C group), low tidal volume group (L group), the high tidal volume group (H group), T-5224 group (low concentration, T1), T-5224 group (high concentration, T2), and DMSO group (D group). In the C group, spontaneous breathing was retained after tracheotomy and intubation without mechanical ventilation. In the L group, the rats were connected to a ventilator after undergoing tracheotomy and intubation, and the tidal volume was set to 7 ml/kg. The peak airway pressure was 10 cmH_2_O, respiratory rate (RR) was 60 breaths/min, inhaled oxygen concentration was 21%, PEEP was 0, and inspiratory/expiratory ratio was 1:2–3, In the H group, the rats were connected to a mechanical ventilator after undergoing tracheotomy; Ventilation parameters refer to the study of Roman et al. [28]. The peak airway pressure of a high tidal volume of 30 ml/kg was approximately 30 cmH_2_O, RR was 60 breaths/min, and inhaled oxygen concentration was 21%. 0. 5 h before the start of the experiment, the C, L, H group were injected with normal saline. Briefly, 0. 5 h before the start of the experiment, the inhibitor + high tidal volume group was injected with c-Fos inhibitor T-5224 (MedChemExpress, USA). The concentrations of inhibitors were 0. 3 mg/kg (T1 group) and 3 mg/kg (T2 group), respectively; meanwhile, the tidal volume was 30 ml/min, the RR was 60 beats/min, and the inhaled oxygen concentration was 21%. In the D group, the tidal volume was set to 30 ml/kg, and DMSO was injected 0. 5 h before the start of the experiment. Mechanical ventilation or spontaneous breathing was observed for 3 h. Approximately 1/3 of the initial anesthesia was added every hour. All animals were sacrificed following ventilation, and bronchoalveolar lavage fluid and lung tissues were collected. This experiment was carried out by observing the “Guidelines for the Care and Use of Laboratory Animals” (NIH Publication No. 85-23, 2011) published by the National Institutes of Health. All of the animal procedures are approved by the Animal Experimental Ethical Inspection Form of Guizhou Medical University (approve number: 2001132) and carried out in compliance with the ARRIVE guidelines.

### Hematoxylin and eosin (HE) staining

HE staining was performed to observe the changes in the lung tissue. After the establishment of the model, the rat lung tissue was immediately fixed in formalin solution at 4 °C for 24 h, dehydrated with alcohol, and embedded in paraffin. The sample was cut into uniform slices with a thickness of 4 µm, placed on a cover glass, and then stained with HE reagents (Solarbio, China). After washing with gradient ethanol, the cover glass was sealed with a neutral gel. The morphology of the alveoli was analyzed under a microscope and scored according to the Smith lung pathological injury scoring system [29].

### Lung wet/dry (W/D) method

After the rats were euthanized, the pulmonary tissue was lavaged with PBS, the right lung was ligated, the right upper lung was separated and weighed. The wet lungs were dried at 65 °C for 48–72 h and then weighed again as a dry lung. The wet-dry weight ratio of lung was calculated.

### Expression of inflammatory factors

The concentrations of TNF-α and IL-6 in bronchoalveolar lavage fluid were detected by enzyme-linked immunosorbent assay (ELISA). The kit was purchased from Cusabio Biotech (Cusabio Biotech, Wuhan, China), and the experiment was carried out in accordance with the manufacturer’s instructions. The optical density at 450 nm was measured using a spectrophotometer.

### Western blotting

The expression of c-Fos (9F6, dilution ratio: 1:1, 000; Cell Signaling Technology, USA) and caspase3 (dilution ratio: 1:2, 000; Proteintech, China) protein was detected by western blotting; protein samples were extracted from the lower lobe of the right lung using a radioimmuno precipitation assay buffer, and the protein concentration was determined by bicinchoninic acid assay. The sample was added to the sodium dodecyl sulfate–polyacrylamide gel electrophoresis (SDS–PAGE) Gel pore. Then, cover SDS–PAGE gels with polyvinylidene difluoride (PVDF) membranes after electrophoresis. The PVDF were blocked with 5% milk for 1 h, incubated overnight with the primary antibody, and washed with TBST. After the secondary antibody (IgG, Dilution ratio: 1:5, 000; Proteintech, Chicago, USA) was incubated for 1 h, the PVDF film was exposed to ECL photoluminescence solution.

### Immunohistochemistry (IHC)

IHC was performed to detect c-Fos and Fasl protein expression. The 4 um slices were dried at 60 °C for 1 h and then dewaxed and hydrated for antigen repair. The sections were cooled, blocked with peroxidase, and dripped with c-Fos antibodies (dilution ratio: 1:400). After DAB staining, the slices were dehydrated until the staining disappears and sealed. A microscope (Leica, Germany) was used to observe and photograph stained slides. All representative images were obtained in a × 200 and × 400 visual field.

### TUNEL staining

Apoptosis of lung tissue was detected. Staining was performed using the TUNEL staining kit (Kaiji Biological Company, Nanjing, China) according to the manufacturer’s instructions. The sections were observed under microscope, and the apoptosis rate of lung cells was calculated, 10 visual fields were taken from each section, and the percentage of apoptotic cells in 100 cells was measured in each visual field. The slides were observed and photographed under a microscope. All representative images were obtained in a × 200 and × 400 visual field.

### Statistical analysis

The data are expressed as the mean ± standard deviation (${\overline{\text{X}}}$ ± SD). Analysis of variance was used for comparisons among groups. One-way ANOVA was used for comparisons between experimental groups. For multiple testing, Tukey analysis was performed. GraphPad Prism 8. 3 and ImageJ 1. 51 software were used to drawing and perform statistical analyses. A *P* value of < 0. 05 was considered significant.

## Result

### T-5224 alleviating VILI

**Fig. 1:**
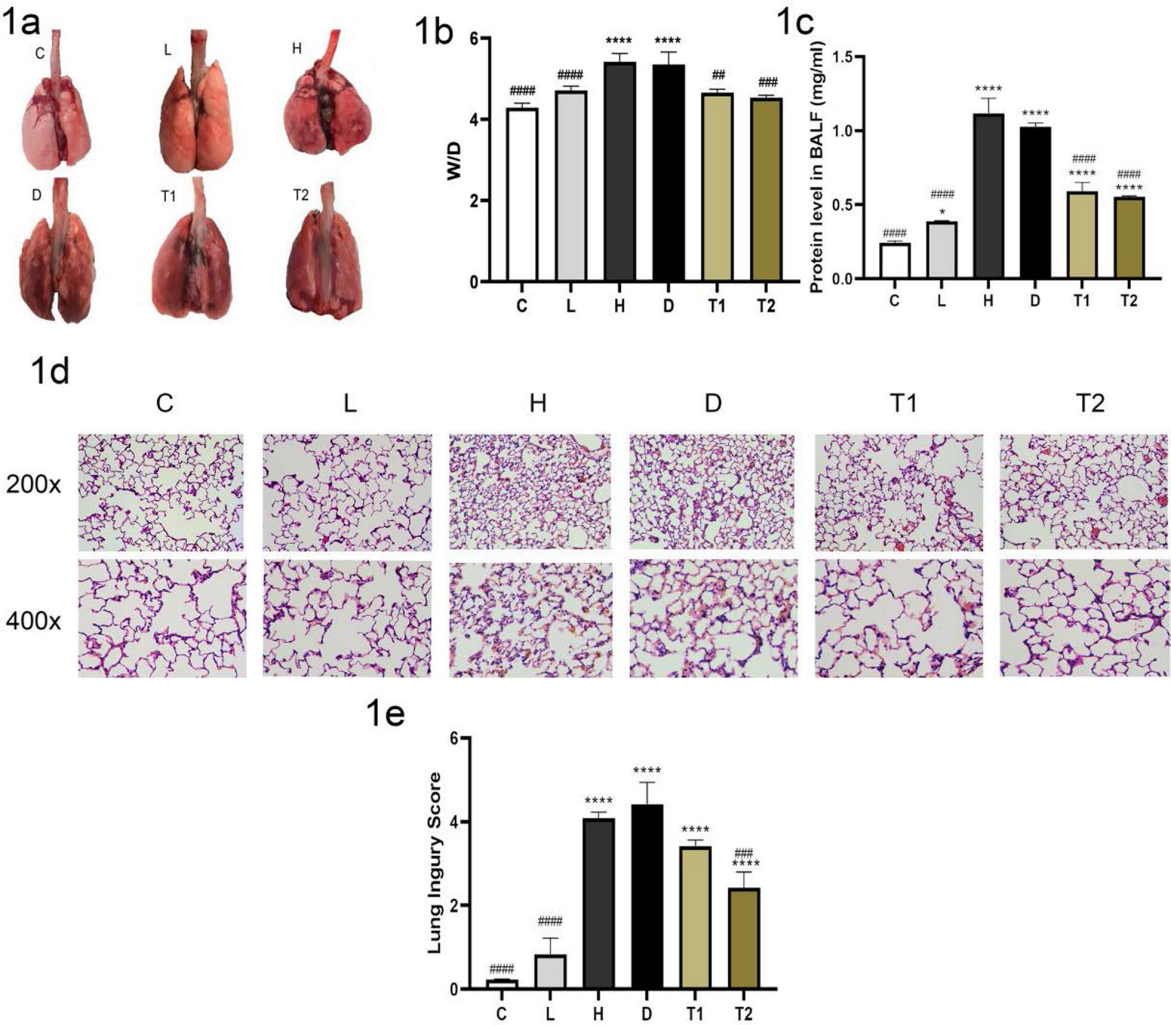
Representative images of gross pathology: severe lung injury occurred in group H and group D, and the lung injury in group T was significantly less than that in group H (**a**); the levels of wet to dry weight ratio (W/D) (**b**); the protein level in BALF (**c**); representative appearances and photomicrographs of hematoxylineosin stained lung sections (magnification × 200 and × 400): severe lung injury occurred in group H and group D, and the lung injury in group T was significantly less than that in group H. Lung histology was characterized by perivascular edema, interstitial and intra-alveolar leukocyte infiltration, and marked heterogeneity in alveolar inflation (**d**); histological sub-scores in experimental groups: lung injury scores were determined based on leukocyte infiltration, exudative edema, hemorrhage, and alveolar wall thickness (**e**). C: Control group, Tidal volum e(TV): 0; L:l ow tidal volume group, VT: 7 ml/kg; H: High tidal volume group, TV: 30 ml/kg; D: DMSO group, TV: 30 ml/kg, Half an hour before making the model, the DMSO was injected; T: T-5224 group, TV: 30 ml/kg, Half an hour before making the model the T-5224 was injected, T1 (0. 3 mg/kg), T2 (3 mg/kg). Data are presented as the mean ± SD (n = 6Rat/group), *P < 0. 0001 versus C;*P < 0. 05 versus C; ####*P* < 0. 0001 versus H; ###*P* < 0. 001 versus H: ##*P* < 0. 01 versus H

The general appearance of lung tissue shows that the lungs of the rats in control group C are pink and normal in appearance. The L group had normal lungs, and there was an absence of bleeding on the surface. The H group exhibited pulmonary hyperemia, obvious edema, and punctate bleeding scattered on the lung surface. Lung tissue injury in the T-5224 treatment group was significantly less than that in the H group; the appearance was brilliant, with slight edema, and a few bleeding spots on the surface (Fig. 1a). In addition, The lung W/D and protein level in BALF of the rats in the H group was significantly higher than that in group C and L (*P* < 0. 05), T group was significantly lower than that in the H group (1b, 1c). After HE staining of lung tissue in each Group, under an optical microscope, the alveolar structures in group C and L were normal; in the H group, interstitial perivascular edema, intra-alveolar and interstitial neutrophil infiltration, alveolar hemorrhage, and hyaline membrane formation were observed. The severity of inflammatory cell infiltration and hemorrhage in group T was significantly lower than those in the H group, and group T had significantly lower lung injury score compared with group H (1d, 1e), C, and L. In summary, the pathological morphology of the lung tissue in group T was significantly improved, and the trend in all indices in group T was opposite to that in the H group.

### T-5224 inhibited the expression of c-Fos protein in lung tissues

**Fig. 2:**
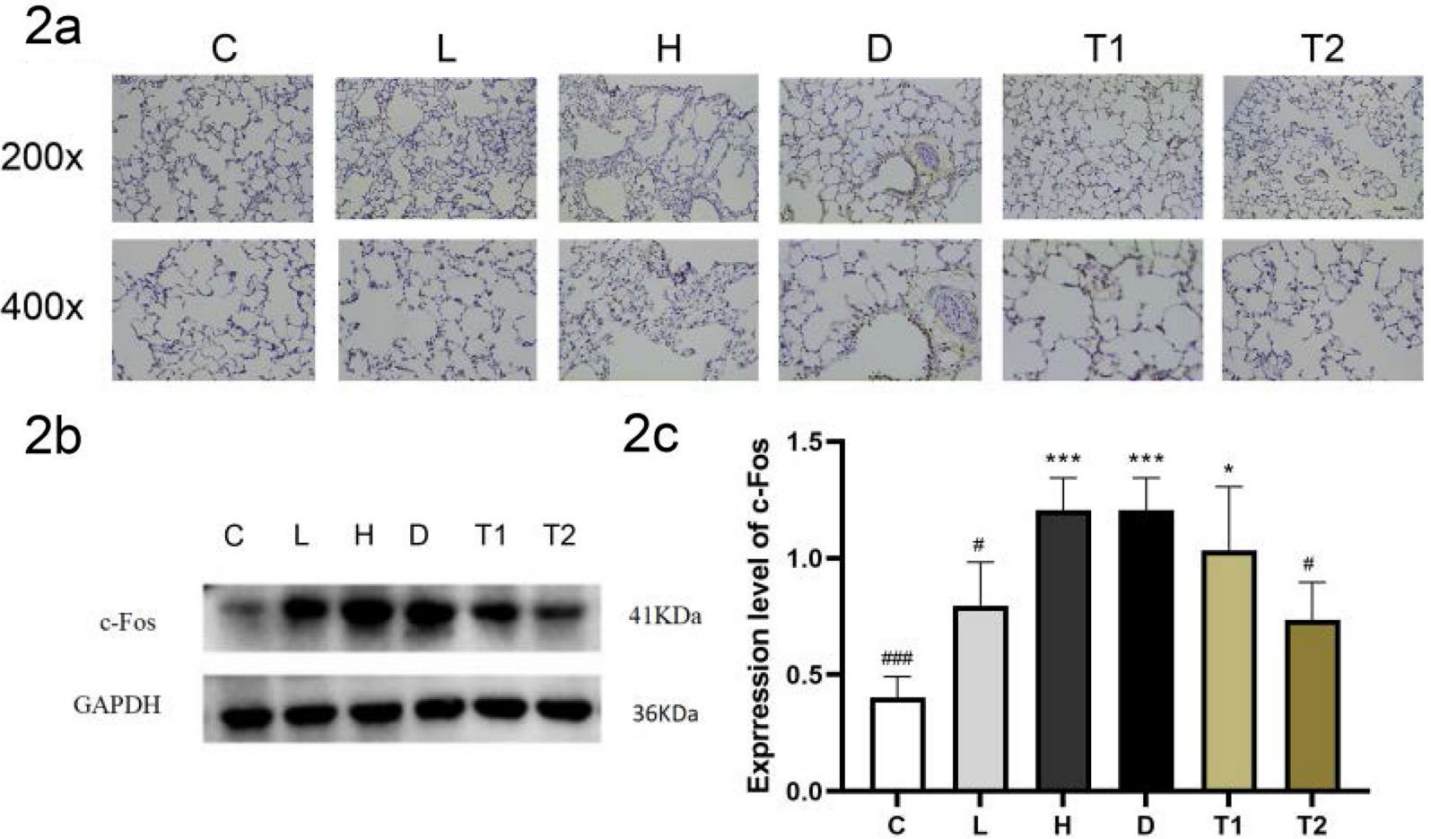
Representative immunohistochemistry images of c-Fos expression of lungs (magnification × 200 and × 400) (**a**); Representative WB images of c-Fos expression of lungs (**b**); densitometry measurements (protein/GAPDH) are mean ± SD (n = 6Rat/ group). The expression of c-Fos in group C and L was significantly lower than that in group H, and the expression of c-Fos protein was mainly in alveolar epithelial cells and bronchial epithelial cells, after the intervention of T-5224, the expression of c-Fos protein decreased significantly (**c**). Data are expressed as the mean ± SD (n = 6 Rat/group) ***P < 0.001 versus C; *P < 0.05 versus C; ###*P* < 0.001 versus H; #*P* < 0.05 versus H

The results of immunohistochemistry and immunoprecipitation showed that compared with the C and L groups, the expression of the c-Fos protein in the H group was significantly increased; and after treatment with T-5224, the expression of c-Fos protein was gradually inhibited with an increase in T-5224 concentration (Fig. 2a–c). These data suggest that T-5224 significantly inhibits the expression of c-Fos in a concentration-dependent manner.

### T-5224 alleviates pulmonary inflammatory response in rats with VILI

**Fig. 3:**
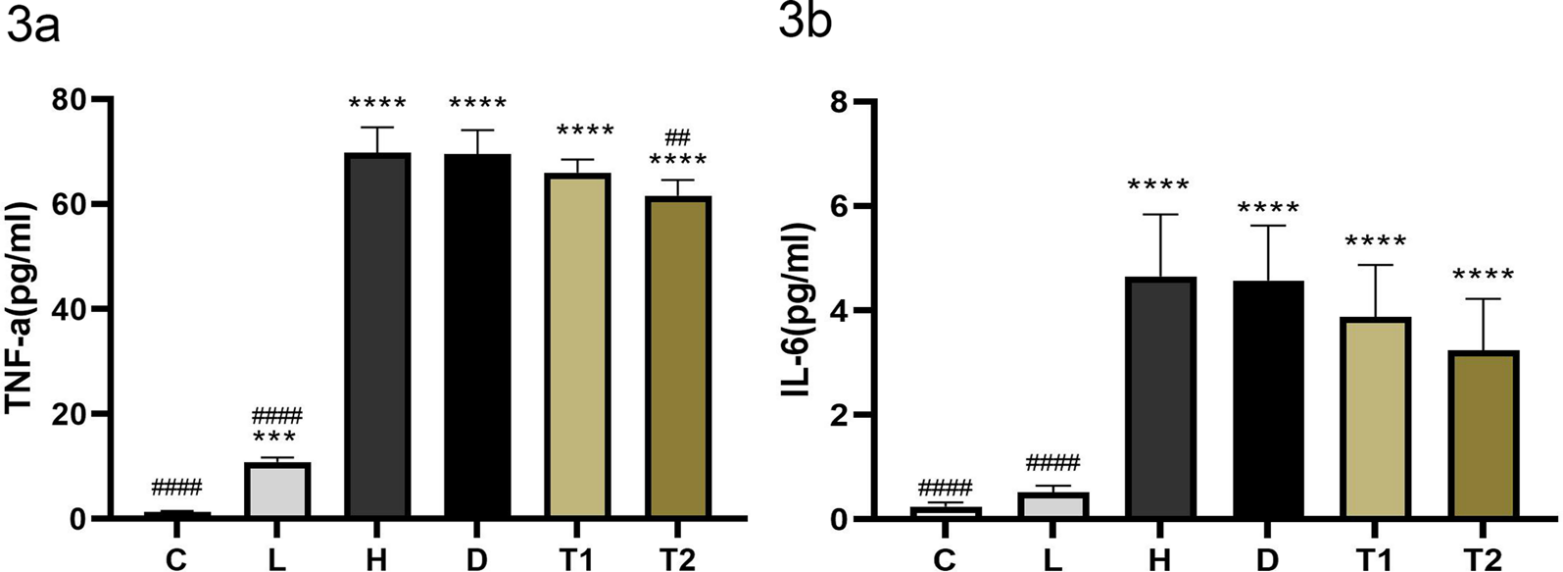
Levels of TNF-a in broncho-alveolar lavage fluid (**a**); levels of IL-6 in bronchoalveolar lavage fluid (**b**), the expression of TNF-a and IL-6 in group C and L was significantly lower than that in group H, after the intervention of T-5224, the expression of TNF-a protein decreased significantly. C: Control group, Tidal volume (TV): 0; L: low tidal volume group, VT: 7 ml/kg; H: High tidal volume group, TV: 30 ml/kg; D: DMSO group, V: 30 ml/kg, Half an hour before making the model, the DMSO was injected; T: T-5224 group, TV: 30 ml/kg, Half an hour before making the model, the T-5224 was injected. Half an hour before making the model, the T-5224 was injected, T1 (0.3 mg/kg), T2 (3 mg/kg). Data are expressed as the mean ± SD (n = 6 Rat/group). ****P < 0.0001 versus C;***P < 0.001 versus C; ####*P* < 0.0001 versus H; ##*P* < 0.01 versus H

We used ELISA method to detect the levels of IL-6 and TNF in BALF to reflect the degree of inflammation in lung tissue. Compared with the C and L groups, the protein expression of TNF-α and IL-6 in the bronchoalveolar lavage fluid of the H group was significantly increased (*P* < 0. 05); after treatment with T-5224, the expression of TNF-α protein decreased significantly, which was significant; the expression of IL-6 protein also decreased; however, the difference was not significant (3a, 3b). Taken together, these data demonstrate that T-5224 alleviates pulmonary inflammatory response in rats with VILI (Fig. 3).

### T-5224 attenuates lung tissue apoptosis in rats with VILI

**Fig. 4:**
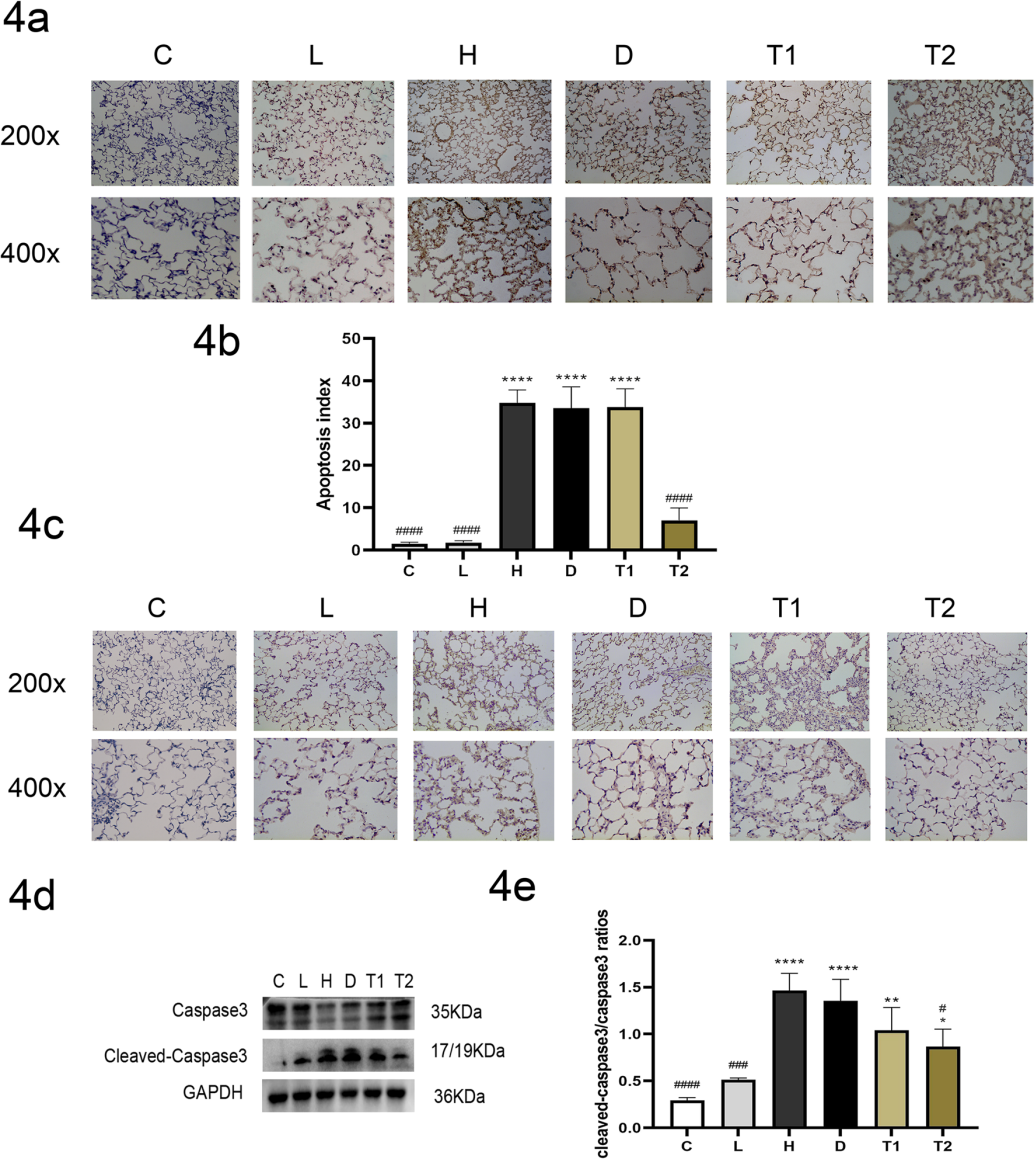
Representative Tunel images of apoptosis (magnification × 200 and × 400): The apoptosis rate is calculated by taking 4 visual fields per slice and measuring the percentage of apoptotic cells in 100 cells in each visual field, The number of apoptotic cells in H group increased significantly, while that in T group decreased significantly compared with H group (**a**, **b**); Representative IHC images of Fasl (magnification × 200 and × 400) Microscopically, Fasl protein was mainly expressed in the cytoplasm, after the intervention of T-5224, the expression of caspase3 decreased significantly (**c**); levels of caspase3 protein in Lung tissue, after the intervention of T-5224, the expression of caspase3 decreased significantly (**d**, **e**). C: Control Group, Tidal volume (TV): 0; H: High tidal volume group, TV: 30 ml/kg; D: DMSO group, TV: 30 ml/kg, Half an hour before making the model, the DMSO was injected; T: T-5224 group, TV: 30 ml/kg, Half an hour before making the model, the T-5224 was injected, T1 (0.3 mg/kg), T2 (3 mg/kg). Data are expressed as the mean ± SD (n = 6 Rat/group). ****P < 0.0001 versus C;**P < 0.01 versus C; *P < 0.05 versus C; ####*P* < 0.0001 versus H; ###*P* < 0.001 versus H; #*P* < 0.05 versus H

Compared with the C and L groups, the cleaved-caspase3/caspase ratio and Fasl of lung tissue in the H group were significantly higher. TUNEL staining results showed that the apoptosis rate of lung tissue in the H group was significantly higher than that in the C and L groups. After T-5224 treatment, the cleaved-caspase3/caspase ratio, Fasl and the apoptosis rate of lung tissue in the 3 mg/kg dose group were significantly lower than those in group H (Fig. 4a–e). Taken together, these data demonstrate that T-5224 can significantly reduce apoptosis in lung tissue, and the specific mechanism may be related to the inhibition of Fas/Fasl expression.

## Discussion

In this study, we found that T-5224 significantly reduced the severity of VILI. T-5224 can significantly ameliorate capillary permeability, and reduce lung tissue inflammation; In addition, the T-5224 can reduce the apoptosis of lung tissue by inhibiting the expression of Fas/Fasl. The main characteristics of VILI are pulmonary inflammation and impaired barrier function. In contrast, mechanical ventilation can cause deformation of the lung cell membrane and receptors, external abnormal mechanical stimulation of the organelles, transformation of biochemical signals into cells, activation of intracellular signal transduction system, and high expression of inflammatory factors, resulting in multiple organ dysfunction syndrome. Mechanical ventilation may also lead to excessive traction on the cells, resulting in damage and repair of some cell membranes. These repaired cells are more prone to apoptosis and can damage the lung tissue barrier system. Apoptosis, also known as programmed cell death, can be triggered by internal or external signaling pathways. In these two pathways, the endogenous pathway is induced by cytotoxic stimulation and then activates the downstream caspases, while the exogenous pathway is triggered by the connection of the death receptor, which directly initiates the activation of upstream caspases. The FasL/Fas-mediated apoptosis pathway belongs to the latter, and Fas is a death receptor located on the cell surface that belongs to the tumor necrosis factor receptor family. The interaction between Fas and its ligand, FasL, can transfer apoptotic signaling and trigger cell apoptosis, the Fas/Fasl pathway has also been shown to play a role in inducing apoptosis in mechanical ventilation-related lung injury [9]. Under normal circumstances, the apoptosis and production of vascular endothelial cells are properly regulated. If a large number of vascular endothelial cells undergo apoptosis in a short period, this will lead to imbalance and endothelial cell barrier dysfunction, resulting in increased vascular permeability and aggravation of pulmonary edema [30–32]. At present, various methods have been used to block the inflammation and apoptosis of VILI, but with little effect. These studies suggest that VILI is not a single inflammatory cytokine disease; it involves various cytokines and apoptosis pathways. Hence, blocking only one factor may not cure this condition.

c-Fos protein binds to the c-Jun protein to form AP-1 to regulate the transcription of various proinflammatory factors and apoptosis genes, which are related to the progression of several diseases. As a c-Fos/AP-1 inhibitor, T-5224 has significant anti-inflammatory effects in many diseases. However, the effect of T-5224 on VILI has not been studied; In this study, T-5224 was injected intraperitoneally in rats exposed to mechanical stress to evaluate its effect on VILI. c-Fos is a proto-oncogene that regulates the transcription of many genes. c-Fos protein expression increased under mechanical stress, and c-Fos and c-Jun protein could specifically bind to form heterodimer transcription factor AP-1 through the leucine zipper pathway. AP-1 binds to DNA primarily through basic leucine zipper and participates in the gene transcription of some growth factors and inflammatory factors. AP-1 can also regulate apoptosis by binding to the AP-1 binding sites of the FAS/FASL or BimEL genes [15–18]. Our results are consistent with those of a previous study by Tremblay et al. [28], which reported that c-Fos expression was increased in the lung tissue of patients with VILI. c-Fos may play an important role in the pathogenesis of VILI. Based on the crystal structure of the AP-1 DNA complex and three-dimensional pharmacophore modeling, T-5224, a specific inhibitor of c-Fos/AP-1, was synthesized. Compared with traditional anti-cytokine therapy drugs (such as anti-TNF-α drugs), c-Fos/AP-1 inhibitors are considered ideal drugs that affect the binding of pro-inflammatory cytokine promoter AP-1 motifs to c-Fos/AP-1 during an inflammatory response. c-Fos/AP-1 directly controls the expression of inflammatory cytokines by binding to AP-1 motifs in the promoters of these genes. In addition, c-Fos/AP-1 can also mediate apoptosis by transcriptional regulation of FasL gene expression, thus initiating the external apoptosis pathway. Aikawa et al. [18] showed that oral T-5224 can eliminate the inflammatory response in an arthritis mouse model by inhibiting the expression of IL-1 β and IL-6. They found that the autopsy results of rats and monkeys did not show any side effects after taking T-5224150 mg/kg and 750 mg/kg daily for a month. Ishida et al. [33] confirmed that T-5224 can inhibit the expression of early and late proinflammatory cytokines, thereby reducing the mortality of mice with acute renal injury caused by sepsis. Shinichiro et al. [34] confirmed that T-5224 can attenuate liver injury by reducing inflammation in endotoxemic mice.

In this study, we used high tidal volume ventilation to create a rat model of a VILI. Through this model, we confirmed that acute inflammatory reaction occurred in rat lung tissues during the high tidal volume ventilation, the apoptosis rate of lung tissue significantly increased, and the content of caspase3 and Fasl protein in lung tissue significantly increased, resulting in lung pathological injury and the formation of pulmonary edema. We found that IL-6 and TNF-a expression was increased in the lung tissue of VILI rats. IL-6 is a multifunctional cytokine produced by several cell types. Experimental evidence supports the role of IL-6 in promoting vascular endothelial cell permeability and inflammation, which potentially aggravates acute lung injury [35–37]. TNF-α is produced by activated monocytes and macrophages, which are called inflammatory initiators. It has a wide range of functions, such as neutrophil activation and vascular endothelial cell aggregation and adherence, causing damage to vascular endothelial cells and increasing the permeability of endothelial and epithelial cells. In addition, we found that the apoptosis of lung tissue induced by c-Fos is related to the upregulation of Fas/Fasl expression. c-Fos/AP-1 can bind to the AP-1 motif of the Fasl gene and enhance the expression of Fasl. Fas/Fasl, as the key pathway to induce apoptosis, finally induces lung tissue apoptosis. Our study showed that c-Fos protein expression was significantly increased in VILI rats. When T-5224 was used, c-Fos protein expression was significantly decreased, and pulmonary inflammation and apoptosis were significantly reduced. T-5224 can reduce the inflammatory response by regulating the expression of a variety of inflammatory factors, reduce lung apoptosis by regulating the expression of Fas/Fasl, and attenuates mechanical stress-induced lung injury in rats.

The increased expression of c-Fos under mechanical stress may be related to the tension-sensitive cation channel (TPR). The change in airway pressure can activate the TPR located in the cell membrane, thus opening the intracellular mechanical signal transduction channel. TPRV4, a subtype of this channel, is a nonselective permeable cation channel that is extensively dispersed in the nervous system, lung, skin, and other systems in the body. Pairet et al. [38] have confirmed that inhibition of TRPV4 can reduce stretch-induced inflammatory cell responses and pulmonary barrier dysfunction during mechanical ventilation. TRPV4 is activated by mechanical stress, which opens the channel to cause calcium influx. Calcium ions act as second messengers to further activate the intracellular signal transduction molecules, such as PKC and ELK, which may eventually induce the expression of c-Fos. However, the specific mechanism still needs to be studied further [39].

This study has two limitations. First, we were unable to evaluate whether T-5224 specifically combines with c-Fos/AP-1 in lung tissues;hence, further studies are needed to clarify whether T-5524 restrains the activation of AP-1 in lung tissues induced by mechanical stress. Second, our study was limited by the administration time and concentration of T-5224. In the clinical environment, the treatment of VILI typically begins a few hours or more after disease onset. In this study, T-5224 was administered prior to modeling. In addition, a maximum T-5224 concentration of 3 mg/kg was used; the therapeutic effect of a higher concentration of T-5224 on the lung tissues of VILI patients needs to be studied further. These two limitations need to be addressed in future research.

In summary, our data show that c-Fos has proinflammatory and proapoptotic effects in patients with VILI, which is a factor that induces inflammation in VILI. Strong expression of c-Fos can cause inflammation, apoptosis, edema formation, and lung injury. The c-Fos/AP-1 inhibitor T-5224 significantly attenuates VILI, which may be due to the inactivation of many inflammatory factors caused by c-Fos/AP-1 signal interference. c-Fos/AP-1 inactivation can also reduce lung apoptosis by inhibiting the expression of Fas/Fasl. This study revealed the important function of c-Fos in VILI. Therefore, c-Fos may be an effective target to slow down the progression of VILI. Future studies are warranted to explore the mechanism of enhanced expression of c-Fos in VILI and the specific mechanisms by which c-Fos promotes inflammation and apoptosis**.**

## Data Availability

The datasets used and analyzed during the current study are available from the corresponding author on reasonable request.

## Abbreviations

VILI: Ventilator-induced acute lung injury
MV: Mechanical ventilation
SD: Sprague–Dawley
SPF: Specific-pathogen-free
HE: Hematoxylin and eosin
IHC: Immunohistochemistry
TNF-α: Tumor Necrosis factor-α
ELISA: Enzyme-linked immunosorbent assay
IL-6: Interleukin-6
W/D: Wet/dry
TPR: Tension-sensitive cation channel
